# A Multifunctional Smart Meter Using ANN-PSO Flux Estimation and Harmonic Active Compensation with Fuzzy Voltage Regulation

**DOI:** 10.3390/s21124154

**Published:** 2021-06-17

**Authors:** Edson A. Batista, Moacyr A. G. de Brito, João C. Siqueira, Jeandro C. Dias, Raphael C. Gomez, Maurilio F. R. Catharino, Matheus B. Gomes

**Affiliations:** 1 Graduation Program in Electrical Engineering, Federal University of Mato Grosso do Sul—UFMS, PPGEE, Campo Grande 79070-900, MS, Brazil; edson.batista@ufms.br (E.A.B.); joao@engtecnologia.com (J.C.S.); matheus.bueno@ufms.br (M.B.G.); 2 Electrical Engineering Department, Dom Bosco Catholic University, Campo Grande 79117-900, MS, Brazil; jeandroec@gmail.com (J.C.D.); raphaelceni@gmail.com (R.C.G.); 3 FCA US LLC, 1000 Chrysler Drive Auburn Hills, Michigan, MI 48326-2766, USA; maurilio_f_catharino@whirlpool.com

**Keywords:** APF, artificial neural network, bidirectional measurement, fuzzy, FPGA, smart meter

## Abstract

This paper aims to present the analysis and development of a complete electronic smart meter that is able to perform four-quadrant measurements, act as a three-phase shunt active power filter (APF), and control three-phase induction motors by stator flux estimation. A transmission control protocol together with Internet protocol (TCP/IP) communication protocol for the remote access of measurement data is embedded into the application to securely transmit reliable information. An artificial neural network trained with particle swarm optimization is used for stator flux estimation, and a fuzzy logic controller is adopted to regulate the power converter DC bus voltage. The present work gathers knowledge from multidisciplinary fields, and all applied techniques have not been proposed altogether before. All control functions are embedded into a field-programmable gate array (FPGA) device, using VHSIC Hardware Description Language (VHDL), to enhance efficiency taking advantage of parallelism and high speed. An FPGA-in-the-loop cosimulation technique was first applied to prove the control functions’ functionality, and, later, experimental evaluations are conducted to finally prove equipment operation and reliability.

## 1. Introduction

The generation, distribution, and measurement of electricity are some of the main themes studied by scientists and researchers all over the world due to their considerably higher efficiency in processing and transporting over other energy sources. Brazilian electricity scenario remained unchanged for at least a hundred years; however, some factors such as fault detection, remote measurements, fraud, and mainly distributed generation have led to the establishment of intelligent grids, also known as smart grids.

Due to the intense usage of nonlinear loads in industries and companies, the quality of the electric power system is impaired with the more apparent occurrence of harmonics and reactive power flow. Some of the major nonlinear loads are identified as rectifiers, converters, and arc devices that pollute the distribution system [1,2]. Devices such as passive and active filters are widely used for harmonic current mitigation and voltage distortion reduction and can be applied separately or together, forming hybrid filters [3,4,5]. The main difference between these filters is related to their complexity and flexibility of use; nevertheless, both have the goal of eliminating or reducing the harmonic content, achieving reactive power compensation and power factor correction to meet the standards such as IEEE 519, 1547, and IEC 61006. This aids in avoiding the major effects of harmonics in the energy system, which are conductors overheating, equipment failures, resonances occurrence, and the premature aging of components [6,7].

In this sense, the development of a platform—mainly, but not restricted to, 220 V_RMS_ three-phase systems—is proposed. The system’s measurement and control functions are vital to enhancing microgenerators’ power quality for the microgrid current and future scenarios. In addition, this platform has the objective of monitoring, with online web communication and a graphical interface, the bidirectional power flow and harmonic content and providing flux estimation and active harmonic mitigation, forming a multifunctional device (smart meter). Measurements of power flow and harmonic content were performed based on the instantaneous power theory (pq theory), which identifies the harmonic currents injected by nonlinear loads as oscillating power components [8]. In that sense, the proposed system contains an active power filter (APF) not only for the compensation of harmonics but also to compensate for the reactive power flow [9].

A solution for the equipment implementation on systems where certain levels of power quality and speed of response are required can be achieved through rapid prototyping hardware. The usage of reconfigurable logic devices, such as a field-programmable gate array (FPGA), allows that the input data and all the required algorithms are processed in real-time, with concurrent and parallel operation schemes, maximizing system effectiveness.

To provide expert control, with high performance and great robustness, for working with different loads, a fuzzy controller was proposed to control the APF DC bus voltage. The use of fuzzy inference systems is actually present in several areas with outstanding achievements [10,11,12].

A multilayer perceptron artificial neural network (MLP-ANN) was trained via a particle swarm optimization (PSO) algorithm for stator flux estimation. The flux estimation is used for controlling the three-phase induction machines using direct torque control (DTC). The rapid response for the required electromagnetic torque demands associated with the reduced stator flux oscillations is the main advantage of drives using the DTC strategy [13,14,15]. Moreover, the proper choice of switching presents a reduction in the switching frequency and losses reduction. The MPL-ANN was chosen because of its parallel processing ability, speed of response, learning ability, robustness, and great response when dealing with nonlinear systems; in the same sense, PSO is a very interesting alternative for the optimization of problems that fall into complex nonlinear functions (i.e., activation functions in neural networks). The great advantage of the PSO method is the ease of structuring and understanding, as well as the simplicity of implementation [16,17,18,19].

To ensure storage and data security, a web system was developed to receive and send information to the proposed device via a transmission control protocol together with Internet protocol (TCP/IP). The device sends measurement information to the system, and all data are saved on a database. The objective of this web system is the centralization of information from smart meters, as it allows monitoring and data transmission. The web platform was designed so that the server was able to communicate with multiple smart meters in the sense that, through this web system, it is possible to analyze and collect all data sent in one place. This feature is interesting for medium- and long-term analysis.

Some of the main features of the proposed FPGA-based smart meter can be highlighted as monitoring loads and grid power quality, compensating harmonics, increased power factor, and control of induction motors, even in very low-speed situations.

Considering the achievements of the present work regarding the existing literature, we cite no overshoots and zero steady-state error for the four-quadrant measurements. They were achieved with less computation burden than [20] and with less error than [21] because of the correct fixed-point representation with a lower-order filter and better flux estimation than [14], with zero error and only with small oscillations at the waveform peaks, even during speed step changes. In [14], one may verify for the flux space vector (d vs. q flux) low-frequency oscillations in all space vector waveforms, and, thus, the same oscillations are shown in the rotor flux. Verifying [22] and [23], we achieved a lower total harmonic distortion for the grid currents, obeying international standards, and reduced voltage fluctuations at the DC bus with fewer membership functions in the fuzzy inference machine. Finally, the usage of the TCP/IP protocol in the proposed smart meter allows a standardized and popular way to connect the electrical system to the web. In [24], one may verify the tendency to exchange data from the smart meter and the web server, but it is intended only for single-phase low-power systems, whereas the present work deals with three-phase systems.

Therefore, the main advantage of the proposed smart meter consists of having only one device with multifunctional properties as well as instantly monitoring. It is necessary to emphasize that although all concepts are not new in the literature, the present work gathers knowledge from multidisciplinary fields, and all applied techniques have not been proposed altogether before, becoming a challenging task and demonstrating its archival value.

**Figure 1:**
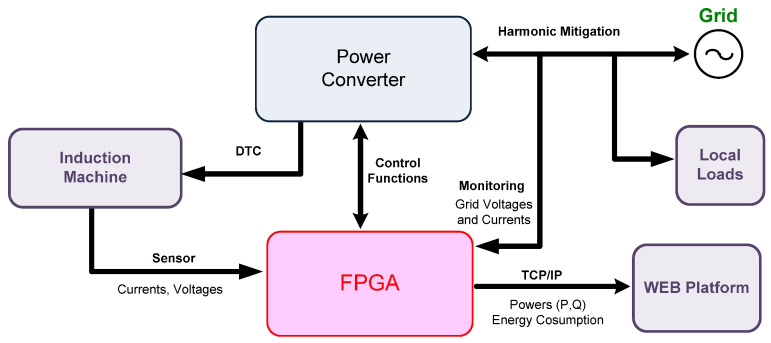
Multifunctional device overall diagram.

Finally, hardware cosimulation and posterior experimental evaluations are conducted to prove the equipment operation and effectiveness. An overall diagram of the proposed device can be visualized in Figure 1, with exchanged data among blocks.

## 2. Material and Methods

### 2.1. Artificial Neural Network and PSO

This paper presents the development of a monitoring platform, in four quadrants, ensuring with a single device the control of induction machines and reduced total harmonic distortion (THD) levels for microgrid operation. Hence, a strategy was developed for estimating the stator magnetic flux through an MLP-ANN trained with PSO. Besides being monitored, the flux is necessary for the DTC strategy.

**Figure 2:**
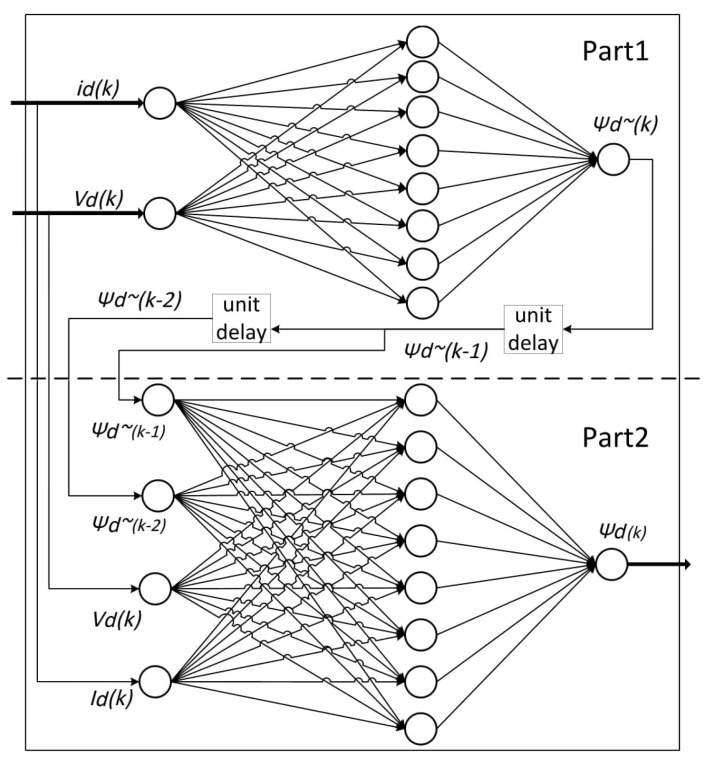
Network structure for estimating the stator flux in the *d*-axis.

Additionally, an MLP network was chosen as it allows the usage of various activation functions (such as “tansig” or “logsig”) that are more effective than simple step functions and enable the network to be applied to nonlinear problem solutions [13]. Two separate, but identical, recurrent network structures were used, the first for estimating the magnetic flux along the *d*-axis and the second for evaluating the magnetic flux along the *q*-axis. The former network structure, which used only information concerning the *d*-axis, can be separated into two parts as outlined. In the first, the stator current (id) and the stator voltage (Ud) were the inputs, and the output was the stator flux Ψd ~ (k) estimation. In the second, the stator flux on the *d*-axis was estimated by considering the previous two estimated fluxes, namely Ψd ~ (k−1) and Ψd ~ (k−2). The former network can be visualized in Figure 2. Another identical neural network estimates the q-axis flux.

Offline supervised training was chosen because the data used for training came from simulations. The simulations were performed using the MATLAB/Simulink platform following the equations found in [25]. These networks were trained to estimate the motor flux from low speed to nominal speed and with nominal torque. The main motor characteristics are: 0.5 HP, 220 V_RMS_, 60 Hz, 4 poles, a stator resistance of 0.43 Ω, a rotor resistance of 0.82 Ω, a stator inductance of 2 mH, a rotor inductance of 2 mH, a mutual inductance of 0.69 mH, and an inertia of 0.089 kg∙m^2^. The used stopping criteria were an acceptable error of 0.0001 (between the estimated and real flux) or failing on achieving a better result over 50 times.

The network training was performed using the PSO strategy, which emerged from experiments with algorithms that mimic the social behavior observed in flocks of birds, shoals of fish, and groups of human beings [17]. PSO is based on the social cognitive hypothesis that each individual in a population has their own experience and is able to estimate the quality of that experience. However, because the individuals are part of a social group, they also possess knowledge about their neighbor’s behavior [18,19]. Analysis has shown that the learning process depends partly on the unique experiences of the individual and partly on the experiences of neighbors, implying that decision-making is a function of the past performance of the individual and those of some neighbors. Cultural adaptation depends on three principles [18,19]: (i) self-evaluation—individuals have the ability to sense the environment in order to estimate their own behavior; (ii) comparison—individuals use each other as a comparative reference; and (iii) imitation—important for the acquisition and maintenance of abilities. Individuals with the capacity to evaluate, compare, and mimic are those that are best able to find a group solution for problems presented by their environment and, therefore, to define global behavior as a result of such interactions.

In the PSO algorithm, the individuals of the population are represented by a number of particles, which constitute a swarm moving around a search space. Variations in their attributes lead to new points in the search space corresponding to movements in that space [19]. Each particle will move in a direction determined by its current position *x_i_*(*k*) and velocity *ν_i_*(*k*), the position *i* that led to its best performance (*pB_i_*) or best fitness so far, and the best overall system performance (*G*). The particle’s velocity may be determined from Equation (1).

(1) $$v_{i}\left(k+1\right)=v_{i}\left(k\right)+\gamma_{1}\left(pB_{i}-x_{i}\left(k\right)\right)+\gamma_{2}\left(G-x_{i}\left(k\right)\right),$$

where coefficients *γ*1 and *γ*2, known, respectively, as the cognitive and social components, are limited from zero to one and indicate the relative contribution of each factor at instant *k*. The position of the particle at the next instant, i.e., *x_i_* (*k* + 1), is determined from its previous position *x_i_* (*k*) and its calculated velocity *v_i_* (*k* + 1) as Equation (2).

(2) $$x_{i}\left(k+1\right)=x_{i}\left(k\right)+v_{i}\left(k+1\right).$$

### 2.2. Smart Systems

As technologies for alternative energy sources are in constant development, bidirectional power flow meters will be needed to constitute one of the main requirements for an intelligent grid. By providing information about the active and reactive power flow, one can identify if the client is receiving or providing power to the system.

It is known that it is possible to use four-quadrant measurements for controlling AC induction motors [26] and permanent magnet DC motors [27], as well as for assistance in controlling three-phase power converters [28]. Therefore, this paper demonstrates the usage of the pq theory to extract the active and reactive average powers and represent them in all four quadrants, as well as for compensating harmonic content.

To obtain the oscillating parts for active and reactive powers, the average power is subtracted from the instantaneous power. These calculated average values can be achieved through digital filters such as the moving average or discretized low-pass filters. In this paper, digital low-pass filters were used because they showed good response and used fewer FPGA logic elements. The transfer function for a third-order low-pass filter, with a unitary damping frequency, is given by Equation (3).

(3) $$H\left(s\right)=\frac{\omega_{c}^{3}}{s^{3}+3\omega_{c}s^{2}+3\omega_{c}^{2}s+\omega_{c}^{3}}.$$

The Tustin discretization method is applied at Equation (3) returning Equation (4). The Tustin method [29] was adopted once the s-plane was mapped into the entirely z-plane and the frequency response could be easily verified in the w-plane (complex digital representation of an equivalent of the s-plane).

(4) $$H\left(z\right)=\frac{k_{0}z^{3}+k_{1}z^{2}+k_{2}z+k_{3}}{z^{3}+k_{4}z^{2}+k_{5}z+k_{6}}.$$

**Figure 3:**
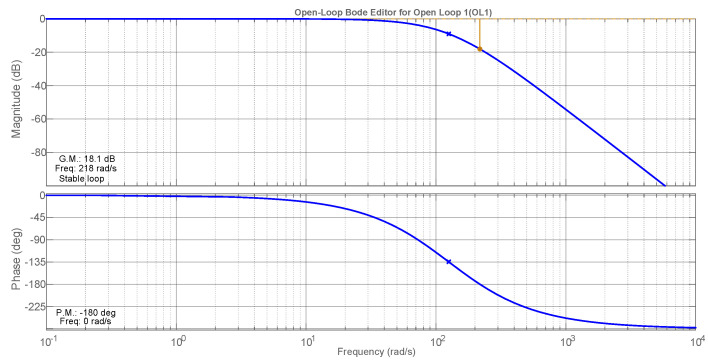
Low-pass filter frequency response.

Through Equation (4), it is possible to describe the filter into a VHDL code, where the coefficients were implemented with a fixed point (43 bits/1 signal bit and 40 bits for the fractional part), and the filter was implemented through the direct representation of its difference equation according to Equation (5), using registers, multipliers, and adders. Table 1 summarizes the filter coefficients considering an acquisition frequency of 30 kHz and a cut off frequency (*ω_c_*) of 20 Hz. The acquisition frequency is the same as the power converter switching frequency to simplify the synchronization procedures among the analog-to-digital converters and the pulse-width modulator. Figure 3 depicts the filter frequency response, with −60 dB/dec and a flat response to maintain the integrity of the DC value.

(5) $$y\left(k\right)=k_{0}u\left(k\right)+k_{1}u\left(k-1\right)+k_{2}u\left(k-2\right)+k_{3}u\left(k-3\right)-k_{4}y\left(k-1\right)\;-k_{5}y\left(k-2\right)-k_{6}y\left(k-3\right),$$

where *y* is the output; *u* is the input; *k* is the current sample; and *k*− 1, *k* − 2, and *k* − 3 are one, two, and three sample delays.

As mentioned, the theory used for detection and compensation is known as the instantaneous active and reactive power theory, or simply, pq theory. It is based on instantaneous values from three-phase systems with or without neutral wires. It is also valid during transients or steady state. The pq theory consists of a Clarke transformation, which converts time-domain signals as voltages and currents from a natural three-phase coordinate system (*abc*) into a stationary two-phase reference frame (*αβ*_0_) [30]. Therefore, it allows active *p* ($v_{\alpha }i_{\alpha }+v_{\beta }i_{\beta }$), reactive *q* ($v_{\beta }i_{\alpha }-v_{\alpha }i_{\beta }$), and zero-sequence *p*_0_ ($v_{0}i_{0}$) powers, and their oscillating counterparts $\tilde{p}$ and $\tilde{q}$ to be examined distinctly and instantaneously. Equation (6) defines the compensation currents ($i_{\alpha }$) and ($i_{\beta }$) from the designated powers, and Equation (7) defines the compensation currents to be injected to the grid by means of the inverse Clark transform, considering power invariability.

(6) $$\left[\begin{array}{c} i_{\alpha }\\ i_{\beta } \end{array}\right]=\frac{1}{v_{\alpha }^{2}+v_{\beta }^{2}}\left[\begin{array}{cc} v_{\alpha } & -v_{\beta }\\ v_{\alpha } & v_{\alpha } \end{array}\right]\left[\begin{array}{c} -\tilde{p}+p_{0}\\ \tilde{q} \end{array}\right].$$

(7) $$\left[\begin{array}{c} ic_{a}\\ ic_{b}\\ ic_{c} \end{array}\right]=\sqrt{\frac{2}{3}}\left[\begin{array}{ccc} \frac{1}{\sqrt{2}} & 1 & 0\\ \frac{1}{\sqrt{2}} & -\frac{1}{2} & \frac{\sqrt{3}}{2}\\ \frac{1}{\sqrt{2}} & -\frac{1}{2} & -\frac{\sqrt{3}}{2} \end{array}\right]\left[\begin{array}{c} -i_{0}\\ i_{\alpha }\\ i_{\beta } \end{array}\right].$$

**Figure 4:**
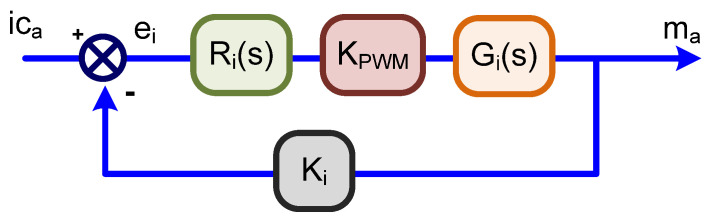
Current control loop—PhaseA.

A current controller is necessary for imposing Equation (7) into the grid through pulse-width modulation (PWM) at the APF converter. The small-signal average modeling was applied to obtain the main transfer functions of the adopted three-phase voltage source inverter (VSI) with inductive filters for grid connection. This controller is based on the average current mode control [31], and the adopted current compensator (*Ri(s)*) has a pole at the origin, a zero, and another pole at a higher frequency (for reducing switching frequency noise). One current control loop [32,33] is depicted in Figure 4, once the others are similar with only 120 degrees of phase-shift. Some parameters are vital, such as the inductance (*L_c_*) value, obtained from Equation (8), which is based on the maximum output current ripple (Δ*i_LC_*), switching frequency (*f_s_*), and grid voltage peak (*V_gridpeak_*).

(8) $$L_{c}=\frac{V_{dc}-V_{gridpeak}}{\Delta i_{Lc}f_{s}}.$$

The APF transfer function is defined in (9), where *V_dc_* is the capacitor DC bus voltage and *rL_c_* is the filter resistance.

(9) $$G_{i}\left(s\right)=\frac{i\left(s\right)}{m\left(s\right)}=\frac{V_{dc}}{sL_{c}+rL_{c}}.$$

The transfer function for the current compensator is given by (10), where *ω_z_* and *ω_p_* are the zero frequency and pole frequency, respectively.

(10) $$R_{i}\left(s\right)=k_{Ri}\frac{s+\omega_{Z}}{s\left(s+\omega_{P}\right)}.$$

To determine (10), one must analyze the open loop transfer function (OLTF), given by (11).

(11) $$OLTF\left(s\right)=R_{i}\left(s\right)K_{i}K_{PWM}G_{i}\left(s\right).$$

**Figure 5:**
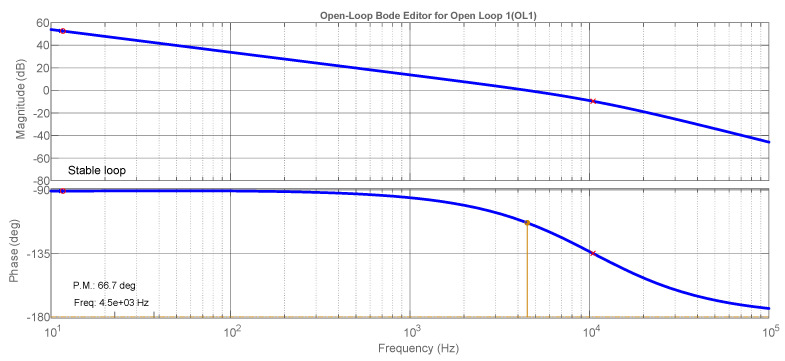
Bode diagrams for the compensated open-current loop.

This control is tuned based on the Bode diagrams of modulus and phase using the criteria of crossing-over frequency and phase margin [31]. The Bode diagrams are presented in Figure 5, where one can observe the compensated characteristics as 4.5 kHz of crossing-over frequency and almost 67 degrees of the phase margin, guaranteeing system stability. The main APF parameters are summarized in Table 2. Tustin was used as a discretization method with a 30 kHz sampling frequency.

Besides the current loop, the voltage loop is also necessary because it keeps the capacitor voltage at a constant DC value [32,33]. In this work, a capacitance value of 365 μF was chosen for reducing the voltage ripple at the DC bus. The voltage loop is controlled through a fuzzy inference system mainly because of the fuzzy characteristics in dealing with nonidealities and plant perturbations [34,35].

**Figure 6:**
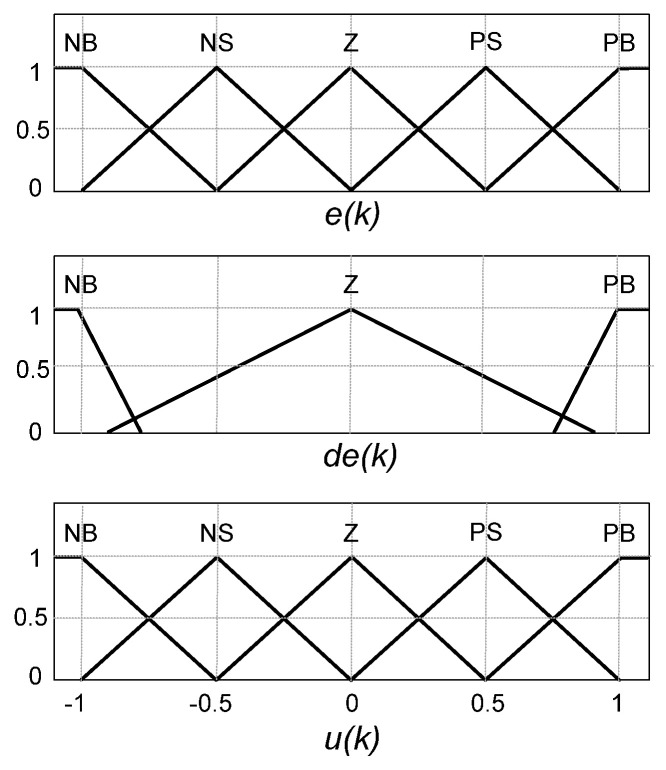
Membership functions for the voltage control loop.

The inference method applied in this controller was Mamdani’s max-prod [36]. The membership function labels are NB, NS, Z, PS, and PB, representing negative big, negative small, zero, positive small, and positive big, respectively. We chose the triangular membership functions (TMF) because of their simplicity and ease of implementation. In this paper, the error (*e(k)*) and its variation (*de(k)*) were selected as input variables. The error is defined as the difference between the DC link reference voltage, which is 400 V, and the capacitor actual voltage. The MFs for *e(k)*, *d(e(k))* and fuzzy output (*u(k)*) are shown in Figure 6. At first, it was adopted five MFs for *de(k)*, and because of that, small variations in the error are considered to generate undesirable noises. Therefore, it was decided to decrease and modify the functions in such a way that, in addition to decreasing the number of rules and, consequently, the hardware usage, it would be possible to eliminate the noise and preserve the action of the controller on sudden variations.

Table 3 summarizes the rules considering the MFs and the output *u(k)* according to the inputs *e(k)* and *de(k)*. The defuzzification method, obtained through Equation (12), consists of calculating the center of the area (COA) after applying Mamdani’s max-prod inference method.

(12) $$u\left(k\right)=\frac{\sum^{\;}u\left(u\left(k\right)\right)\;\;u\left(k\right)}{\sum^{\;}u\left(u\left(k\right)\right)},$$

where *u(u(k))* is the MF value of the discrete element of output value *u(k)*.

**Figure 7:**
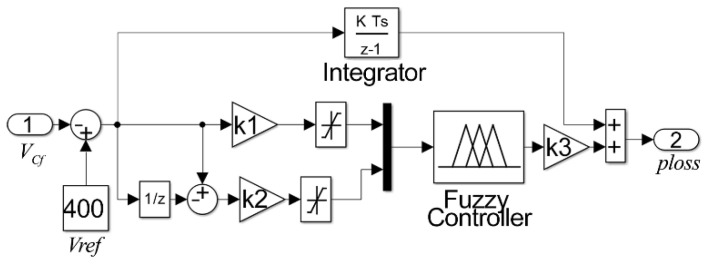
Fuzzy controller for the voltage control loop.

The overall controller configuration integrates the error and sums it after the fuzzy inference machine, therefore eliminating any steady-state error. The integrative term appears in parallel with the inference machine performing a fuzzy PD + I controller; in such a configuration, the zero steady-state error is obtained by the usage of the integrative term, and the inference machine speeds-up the controller response. The explained configuration is shown in Figure 6, where coefficients k, k1, k2, and k3 are chosen as 100, 1, 0.01, and 5, respectively. The aforementioned coefficients were determined throughout extensive simulation analysis. The control signal obtained finally with the configuration of Figure 7 is the power lost in the converter stage, called ploss. This signal is used in addition to the other power compensation signals ($-\tilde{p}+p_{0}$) in Equation (6) to create the compensating currents according to Equation (7).

The communication system was implemented through TCP/IP protocol to enable remote access for monitoring data with a web interface. Hence, access control is a need to secure and ensure the information integrity from the smart meters in a way the user will only have access to these equipment/data through his own profile, and all the information stored in the database allows energy quality analysis.

**Figure 8:**
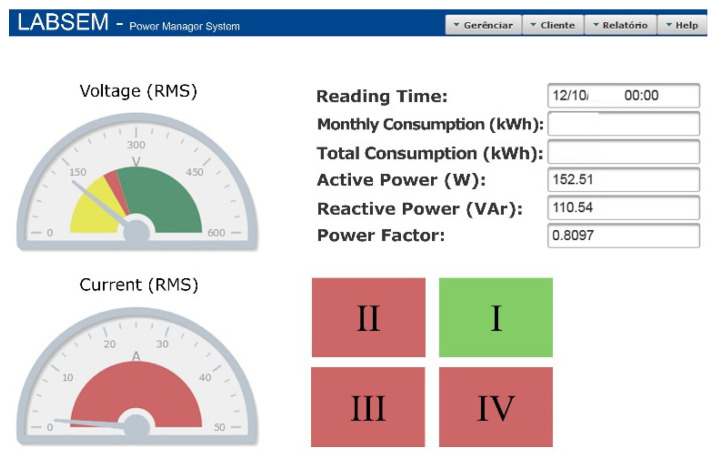
Instantaneous measurements: web interface.

Figure 8 shows one of the system’s interfaces, in which the average voltages, currents, consumption, and quadrants are shown. This web system is Java-based and communicates with the equipment using an API socket. Some of the exchanged data are voltages, currents, active and reactive power, and energy consumption.

## 3. Results

The equipment was tested with a bidirectional meter by changing the three-phase loads: first, a predominant resistive load in delta connection for measuring the active power; second, predominant capacitive and inductive loads inserted with the previous load.

**Figure 9:**
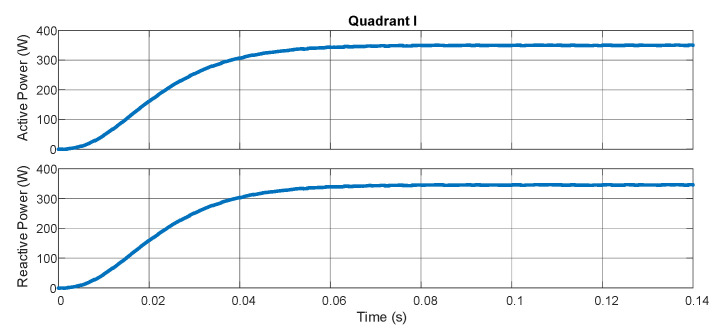
Quadrant 1.

**Figure 10:**
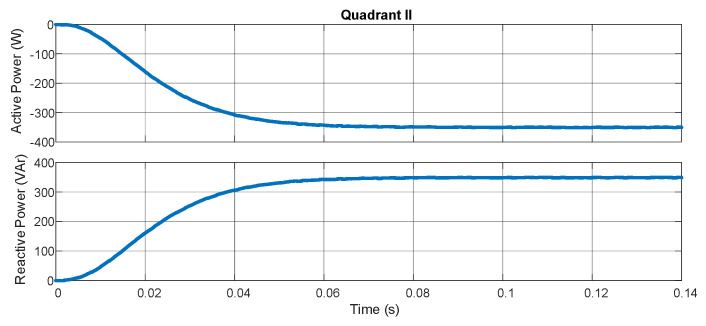
Quadrant 2.

**Figure 11:**
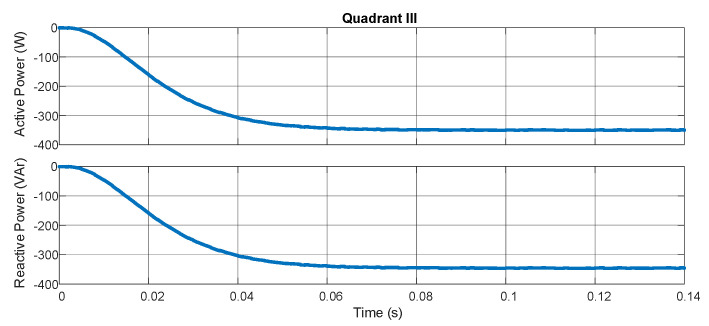
Quadrant 3.

**Figure 12:**
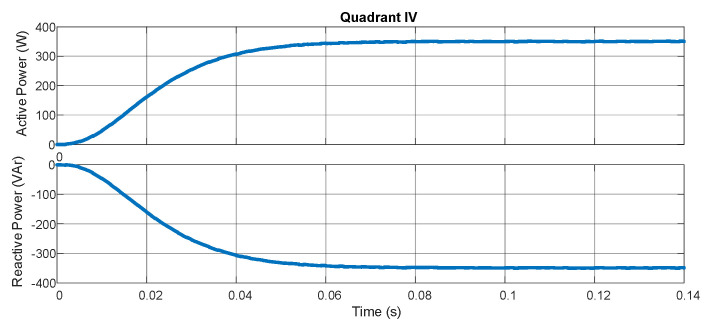
Quadrant 4.

The experimental results characterize the equipment’s ability to measure the bidirectional power flow through pq theory and the digital filters embedded in the hardware. Figure 9 demonstrates the active and reactive powers with the resistive load in series with an inductive load (207 Ω/550 mH for each phase). Figure 10 shows the result for the same loads but with an inverted position of the source while maintaining the position of the sensors. In Figure 11, a capacitive load (12.8 μF) is put in the place of the inductive load. In Figure 12, the load and source positions are inverted again. The power drained by the aforementioned loads, in series connection, are close to 350 W and 350 VAr.

This equipment also has the objective of controlling a three-phase induction motor by estimating the magnetic flux through an MLP-ANN. This strategy was adopted because, at very low speeds, the acquired data from sensors present considerable noise, interfering directly with the motor control. These motors are vastly used in microgeneration systems, and therefore, more optimized usage may interfere directly with power quality and production costs.

The ANN training was performed through the PSO algorithm. The ANN used had two hidden layers with eight neurons in the first and two in the second. PSO was in charge of computing and storing the weights. As explained before, the ANN inputs were voltages, currents, and delayed fluxes from the stator in both the *q*- and *d*-axes. The objective function was based on the error; however, minimizing its absolute values was necessary, according to Equation (13). The major advantage is a reduction in computational cost and training time. Due to a large amount of input data, training algorithms such as Levenberg–Marquardt (LM) [37,38] had memory issues, which resulted in a software crash. PSO, in turn, did not present any problems and, even with lower training points, is faster than LM.

(13) $$E\;=\;\;\left|estimed_{flux}-\;ideal_{flux}\right|.$$

**Figure 13:**
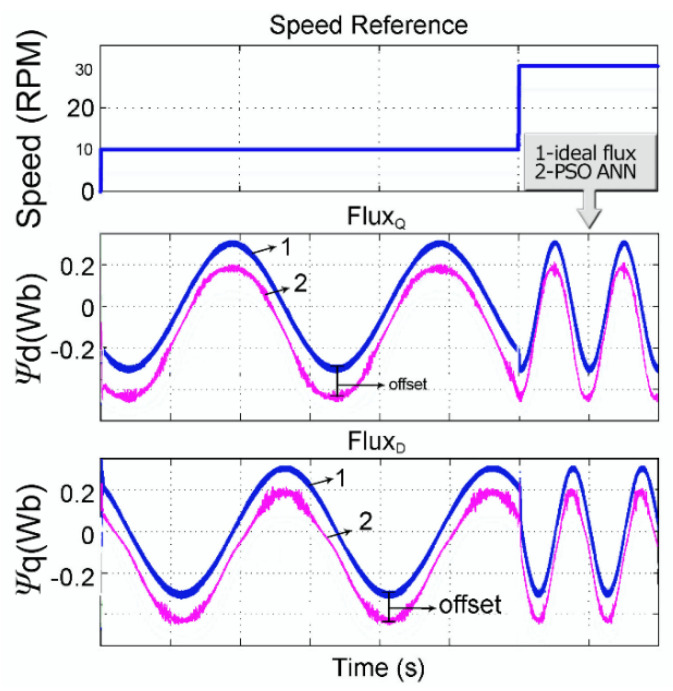
Comparison between estimated (pink) and ideal flux (blue).

**Figure 14:**
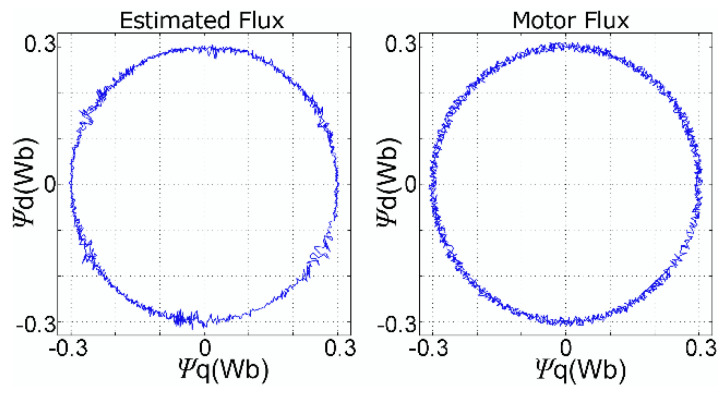
Comparison of fluxes in the *q*- and *d*-axes.

Through simulations in the MATLAB environment, one can check the quality of the estimation and the motor control. Figure 13 shows a comparison between the estimated and optimum flux, in the *q*- and *d*-axes, for the velocities of 10 and 30 rpm controlled by the DTC. An artificial offset was added to facilitate their visualization. Fluxes are also presented in terms of their corresponding axes in Figure 14, where one can verify the quality of the estimated flux, i.e., without error and with small oscillations, even during the step-change.

**Figure 15:**
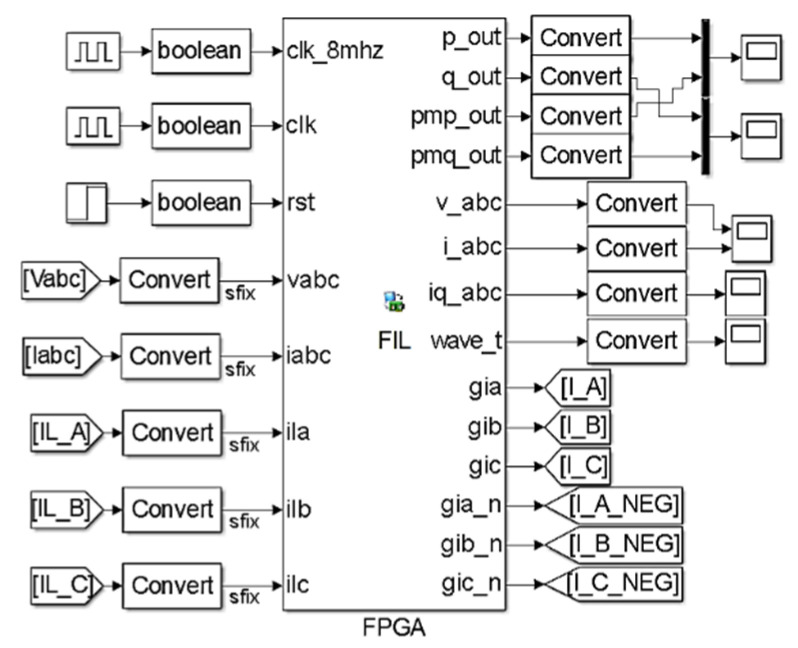
Field-programmable gate array (FPGA)-in-the-loop (smart meter) simulation block.

Hardware described in the VHDL language for testing the control of the APF was implemented. The first part test was taken through FPGA-in-the-loop (FIL), where the hardware is described in VHDL and embedded in the FPGA and the simulation runs on software (MATLAB/Simulink) and hardware (FPGA). The pq theory, current controllers, and PWM were inserted into the FIL as VHDL codes using a 32-bit fixed-point data type for arithmetic purposes. For FIL simulations, the Altera Quartus II was adopted as the compiler, and the system ran at an 8 MHz clock speed. There remains the possibility to generate a customized configuration in case the board lacks predefined configurations and libraries. The FIL block generated by Simulink is shown in Figure 15 with the conversion blocks for sampling rate and data type.

The results obtained for the embedded control module are shown using Cyclone FPGA Altera EP4CE115. The logic occupied only 30% of the device’s total resources, demonstrating the potential of the FPGA and VHDL programming.

**Figure 16:**
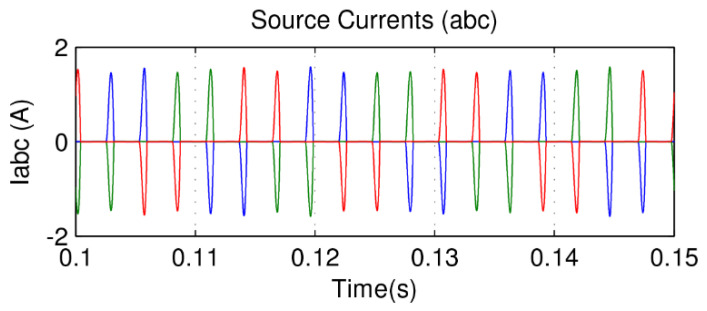
Source current with three-phase induction motor.

**Figure 17:**
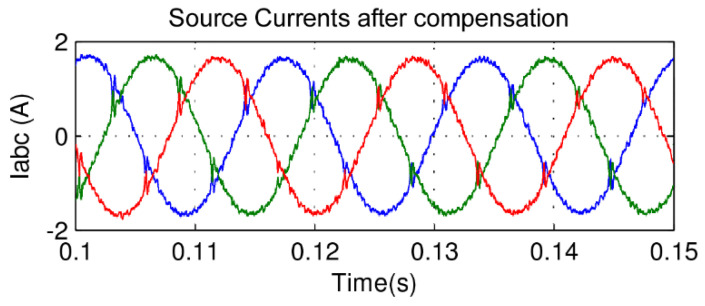
Source current with APF compensation.

In this paper, there is no distortion or unbalance in the voltage source so that the focus of the presented results is on the drained currents. The motor as a load generates considerable distortions in the input source. This is due to the converter input rectifier. As expected, the currents showed a high level of harmonics, as shown in Figure 16. However, Figure 17 shows its currents after the compensation from the APF almost in sinusoidal form, with a total harmonic distortion of 3.5%, far less than the recommendation of 5%.

**Figure 18:**
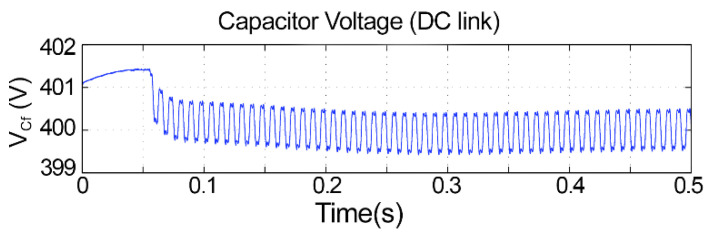
Capacitor voltage with a fuzzy controller.

Figure 18 presents the DC bus voltage with a fuzzy controller. One may note that the voltage fluctuation is within the designed limits, which were 1%, and its average value is 400 V. Its behavior changes after 0.06 s because this is when the equipment finished calculating the average powers and started compensating properly; the small ripple is observed because of the update rate of the algorithm. Because, at all times, the DC voltage is higher than the source voltage and does not exceed the fluctuation limit, even with different loads, the fuzzy controller was validated.

**Figure 19:**
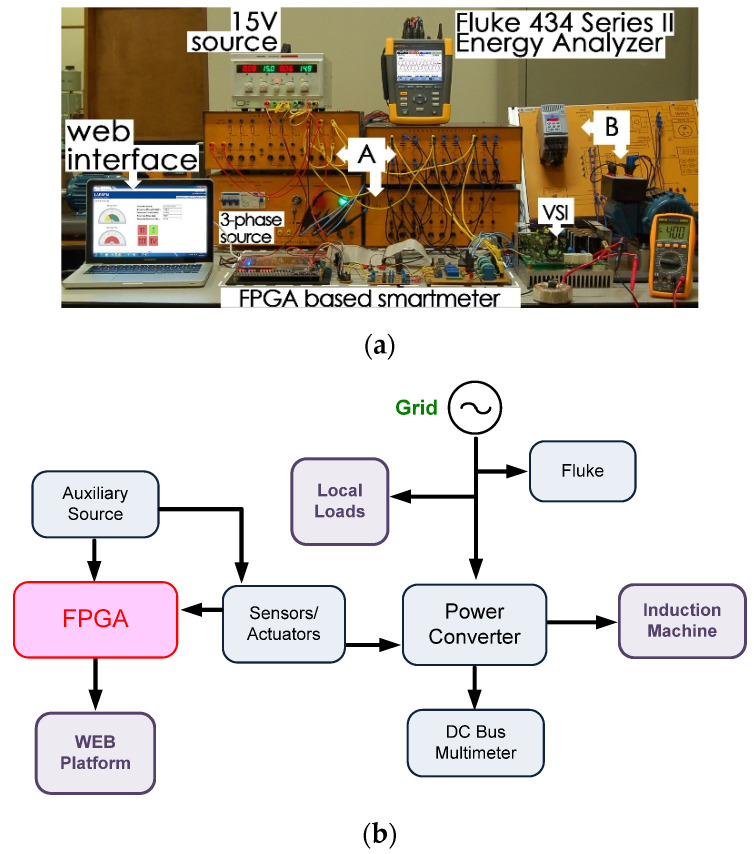
Test bench for smart meter compensation with web interface. (**a**) Experimental setup; (**b**) test bench block scheme.

After FIL validation, the equipment was experimentally verified. The complete test workbench is presented in Figure 19a. In Figure 19b, one may verify the block scheme for test bench comprehension. It comprises the web interface, the FPGA platform, the signal conditioning, a three-phase 220 V_RMS_ source, an induction machine (squirrel gage, 220 V, 60 Hz, 0.5 HP, 4 poles), the variable loads, the Fluke energy analyzer, and the power converter.

**Figure 20:**
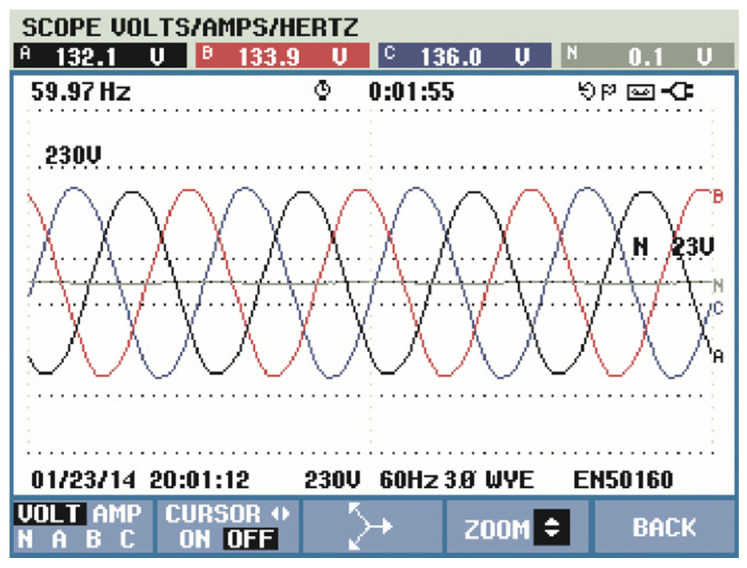
Source voltage measured using the Fluke energy analyzer.

**Figure 21:**
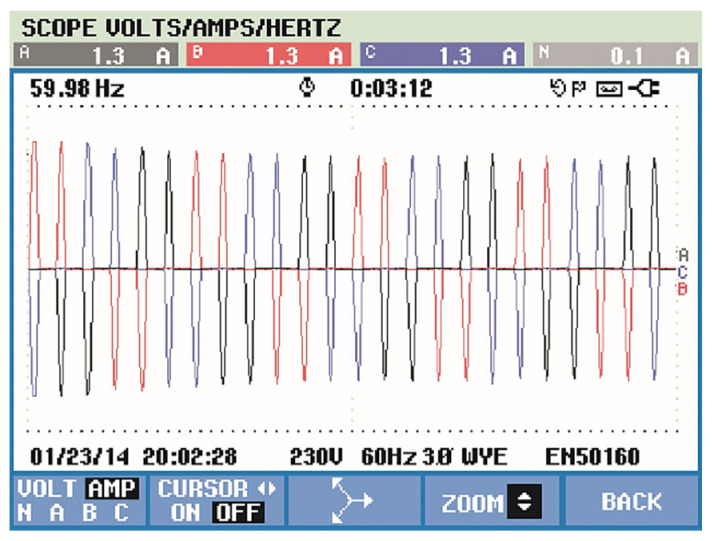
Source current with a nonlinear load (inverter + motor).

**Figure 22:**
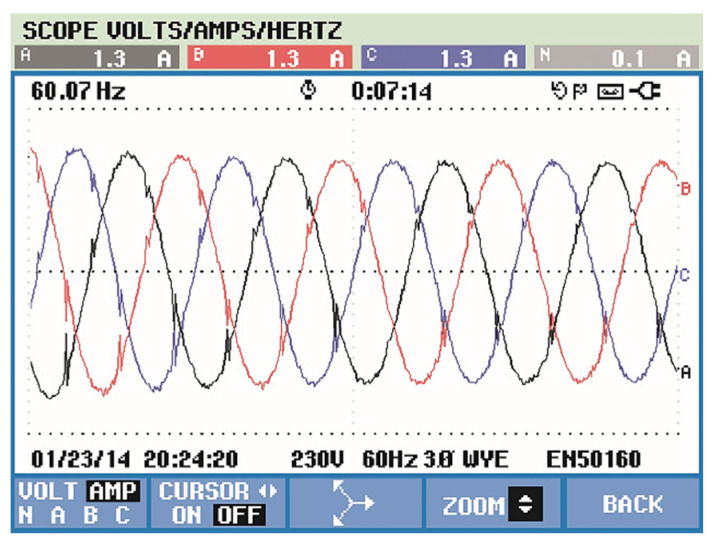
Source current with smart meter active compensation.

The results can be seen in Figure 20, which shows that source voltages are balanced. Figure 21 shows the drained distorted currents when driving the three-phase induction motor. The source currents after harmonic compensation are presented in Figure 22. Therefore, even with a nonlinear load, the multifunctional smart meter is able to detect and compensate for the harmonic currents appropriately.

## 4. Discussions

According to Figure 9, Figure 10, Figure 11 and Figure 12, one may verify the bidirectionality of the proposed smart meter. The measurements showed no overshoots and zero steady-state error with almost no ripple for all the four-quadrant measurements. Observing [20], the present work obtained similar results for the active power measurement with less computation burden once we achieved zero steady-state error with a lower-order digital filter. The authors of [21] showed active power measurements with pq theory with errors in the range of 4%, which is far bigger than our results; this happened most probably because they used a limited fixed-point representation for the calculus. As we used an FPGA, we could choose the correct fixed-point representation for each coefficient.

Additionally, regarding the flux estimation, according to Figure 13 and Figure 14, one may conclude a correct flux estimation, with zero error and only small oscillations at the waveform peaks. The correct estimated fluxes were achieved even during velocity step changes. Verifying [14], it presented for the flux space vector (d vs. q flux) low-frequency oscillations in all space vector waveforms, and, thus, the same oscillations are shown in the rotor flux waveforms. This way, we may verify that our work presents a better flux estimation.

Observing Figure 18, the fuzzy logic controller adopted for the control of the DC bus in a constant average value results in a small ripple of less than 1 V, which represents only a 0.25% fluctuation. Finally, one may observe from Figure 22 the correct compensation of the harmonic content in an almost sinusoidal form, with low harmonic distortion in the range of 3%. Similar findings can be verified in [22], but the total harmonic distortion is in the range of 10%, which is not allowed by international standards. In [23], the authors inserted a fuzzy controller to improve the DC bus regulation, and this helped to minimize the total harmonic distortion to 3.88%, which is similar to our work but still slightly higher. In addition to this, the authors of [22] used nine membership functions, which increases computational burden; in our study, we could achieve the aforementioned improvements with only five membership functions.

Finally, an important verification is that all controller functions are presented separately in the literature, which shows the importance of the proposed work.

## 5. Conclusions

The purpose of this paper is to provide an alternative composed of intelligent monitoring and control systems for use in microgeneration scenarios. This equipment has fundamental properties that meet the concepts necessary for a smart grid, such as four-quadrant measurements, current harmonic mitigation, power factor control, motor control, and data monitoring, via a web system using techniques fully implemented into the VHDL language and embedded processing.

It should be emphasized that the use of PSO training favors a faster and simpler way to estimate the flux using artificial neural networks, assisting in more accurate induction motor control (widely used in some power generation systems) and providing an increase in energy quality. Additionally, the usage of a fuzzy logic controller is an interesting alternative to control the APF DC bus voltage when dealing with different load types. In relation to FPGA and VHDL programming, the logic occupied only 30% of the board’s total resources, demonstrating the potential of the proposed solution.

The results obtained through the energy analyzer are consistent with both software and hardware (FPGA-in-the-loop) simulations and experimental evaluations, serving as the base for the development of more compact, robust, and efficient multifunctional smart meters. The usage of an FPGA as technology to embed the operational logic of the smart meter, as proposed in this work, favors the hardware synthesis of AI algorithms, antifraud systems, the implementation of different communication systems enabling IoT, remote updating capability, and the availability of services to consumers. As FPGA technology favors the execution of algorithms in parallel, the computational resources are attractive to enhance the use of big data and the implementation of advanced measurement infrastructure (AMI), fundamental in the smart grid scenario. The authors aimed to demonstrate a solution that could be used to implement the concepts of Industry 4.0 and IoT in the electricity distribution network, in addition to microgeneration control.

Finally, the present work gathers knowledge from multidisciplinary fields, and all applied techniques have not been proposed altogether before, as we observed from the literature.

## Figures and Tables

**Table 1:** Digital filter coefficients.

Coefficient	Value
*k_0_*	9.13 × $10^{-9}$
*k_1_*	2.739 × $10^{-8}$
*k_2_*	2.39 × $10^{-8}$
*k_3_*	9.13 × $10^{-9}$
*k_4_*	−2.987
*k_5_*	2.975
*k_6_*	−0.9875

**Table 2:** Main active power filter (APF) parameters.

Symbol	Description	Value
*L_c_*	Inductance	700 μH
*V_dc_*	DC bus voltage	400 V
*f_s_*	Switching frequency	30 kHz
Δ*i_LC_*	Current ripple	10%
*V_gridpeak_*	Grid peak voltage	311 V
$K_{i}$	Current sensor	1/10
$k_{Ri}$	Compensator gain	3545
$\omega_{Z}$	Zero	73.2
$\omega_{P}$	Pole	65,820

**Table 3:** Fuzzy rules.

*u(k)*	*e(k)*					
*de(k)*		NB	NS	Z	PS	B
	NB	NB	NB	NS	NS	Z
	Z	NS	NS	Z	PS	PS
	PB	Z	PS	PS	PB	PB

## Data Availability

Not applicable.
